# Design and characterization of a low-cost particle image velocimetry system

**DOI:** 10.1016/j.ohx.2024.e00563

**Published:** 2024-07-24

**Authors:** Frederick Kojo Chaway Acquah, Jeremiah Paul Konadu Takyi, Heather R. Beem

**Affiliations:** Department of Engineering, Ashesi University, 1 University Ave, Berekuso, Ghana

**Keywords:** Particle image velocimetry, Flow visualization, Low-cost, Arduino, Characterization

## Abstract

Particle Image Velocimetry (PIV) is considered the gold standard technique for flow visualization. However, its cost (at least tens of thousands of dollars) can prove inhibitive in its standard form. This article presents an alternative design, leveraging off-the-shelf and open-source options for each key component involved: camera, laser module, optical components, tracer particles, and analysis software. Flow visualization is a crucial technique to connect theory to practice in teaching and researching fluid mechanics. Despite the ubiquity of this field within engineering curricula, many undergraduate institutions globally forego utilizing such equipment, given the barriers to setting it up. The availability of this low-cost alternative (∼$500) that can be built in-house offers a path forward. Characterization was done by visualizing the rotational flow generated by a magnetic stirrer in a cylindrical beaker. The velocity magnitude around the stirrer bar measured by the low-cost PIV system was compared to expected values calculated analytically. The percent difference was between 1–2% when the flow stayed two-dimensional but increased as the flow began developing into more of a 3-D flow. Repeatability varied no more than 6% between experiments. This platform holds the potential for reliable replication across institutions broadly.

## Graphical abstract

**Figure:**
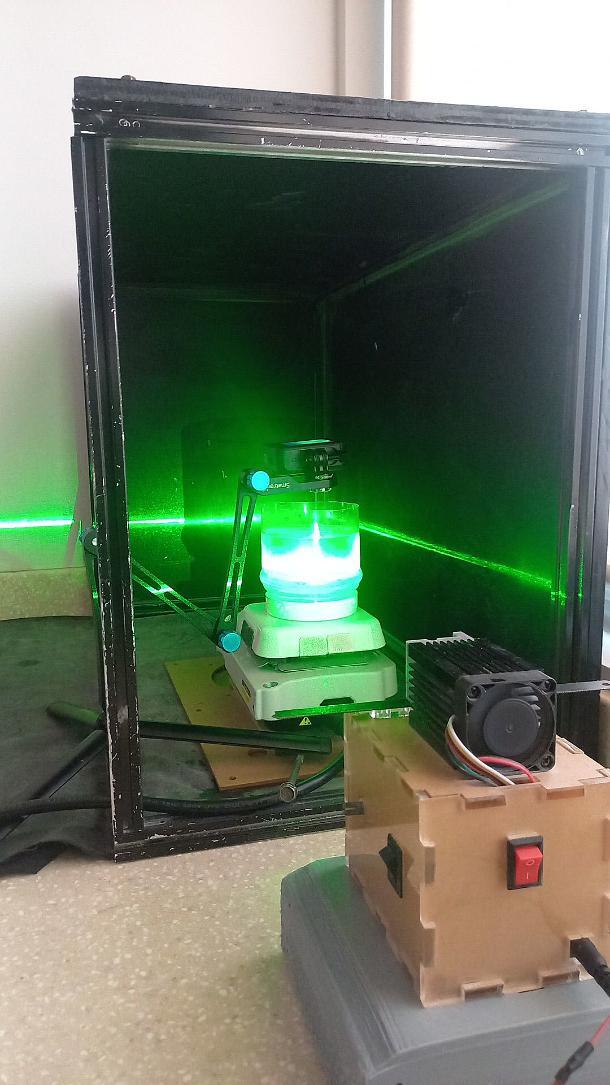

Specifications table

**Table:** 

Hardware name	Low-cost PIV system
Subject area	• Educational tools and open-source alternatives to existing infrastructure
Hardware type	• Imaging tools • Measuring physical properties and in-lab sensors
Closest commercial analog	2D Particle Image Velocimetry system-Microvechttps://piv.com.sg/piv-products/2d-piv-system/
Open-source license	CC BY 4.0
Cost of hardware	$520.5
Source file repository	https://doi.org/10.17632/cgmmttr4bz.2

## 1 Hardware in context

Flow visualization is the category of experimental techniques that enable visual investigation of fluid phenomena. Once visualization has been achieved, one can qualitatively appreciate what phenomena are at play and quantitatively calculate parameters of interest in the flow. These techniques enable basic research, elucidating fundamental mechanisms at play and applied research, informing design decisions [1]. Many different visualization techniques have been developed to support experimental investigation in fluid mechanics. These techniques include dye/smoke discharge, evaporative coatings or tufts of yarn on boundary surfaces, and shadowgraph or schlieren to detect density changes [2].

Particle Image Velocimetry (PIV) is one of the vital flow visualization techniques utilized in experimental work, and many consider it the gold standard technique. It can achieve high resolution spatially and temporally, its correlation-based analysis is considered robust, and it is a non-intrusive approach. The earliest account of a PIV system is believed to be in the 1970 s, when three separate research groups reported accurately and quantitatively measuring the motion of several particles simultaneously using a method known as laser speckle in solid mechanics [3]. They described how a similar approach could be used for measuring fluid flow velocity fields. PIV systems have advanced significantly since then, but they can generally be described as composed of four main components: a laser with optics, tracer particles, a high-speed camera, and analysis software [4], [5]. The high-speed camera captures the motion of several tracer particles illuminated in the flow by a laser. 2-D PIV, which is the type of PIV in focus here, is conducted by first seeding the flow of interest with tracer particles, then illuminating the flow field using optics to form the laser light into a thin sheet, pulsing the laser in synchrony with a high-speed camera. As a test object moves through the field of interest, the PIV system captures the resulting flow field. The series of images are uploaded into the software. Each image is broken down into interrogation windows, and the group of particles captured in each window is compared from one-time step to the next. The velocity vector likely to describe the motion of the group of particles in each window is generated utilizing cross-correlation. The vectors generated for each window collectively comprise the vector field for the entire Image. This vector field information calculates various parameters of interest, such as velocity, pressure, and vorticity across the field of view.

Several established companies offer PIV systems as a complete package. These costs are tens to hundreds of thousands of dollars, driven by the cost of the hardware components and the proprietary software involved. The authors’ search for commercial options that fall on the cheaper end of the spectrum led to the following two, which are based on information stated on their websites and quotes received. Microvec's most basic 2D-PIV system, which comes with a high-speed camera, hollow glass sphere tracer particles, custom software, and a 1 W laser integrated with optics, costs around $15,000 [6]. Optolution's 2D-PIV system uses a 5 W laser integrated with optics, an OPTO cam camera with a band filter (specified region of interest is 300 by 250 mm), tracer particles, and the open-source PIVLab software. This system goes for around $9,000 [7].

Over the years, many researchers have pursued means of reducing the cost of PIV systems. In the early 2000′s, this was mainly through the use of alternative illumination sources, replacing the laser system with white light [8] or LEDs [9], [10], [11]. Around the 2010′s, use of laser pointers began being documented as well [12], [13]. This period also saw the exploration of alternative imaging sources as lower-cost high-speed cameras came onto the market [14], [15], [16], [17]. This included the beginning of the use of smartphone cameras as the imaging source [18], [19], [20]. From the 2020′s, exploration of other alternatives has been seen, such as developing mobile apps to perform analysis [21], [22] and improving on open-source versions [23].

Despite these advances, many systems remain out of reach in the authors’ context. Some work which was described as “low-cost” still quoted a total cost in the thousands of dollars [14], [15], [16]. Indeed what was described as a “low-cost” camera cost $3,000 [13]. Of the options identified, the cheapest one, based on costs reported, was about $700 [12], although the camera used is no longer available on the market. Although these are cheaper than the standard packages produced by companies, an even lower-cost option in the range of hundreds of dollars is needed. The authors sought to develop a system that would work in their home institution in Ghana and across Sub-Saharan Africa, where the economic status of many countries [24] tends to limit the budgetary allocations available for teaching and learning equipment. The authors aimed to create a reliable PIV system that would cost less than $1,000 and be replicable by universities across the region.

The system developed here, which may hereafter be referred to as “the PIV system”, contains the essential components of a standard PIV system, as described above. Off-the-shelf and open-source options were identified for each of these. It was decided to leverage a laser module (chosen for its higher light intensity and ability to be programmed to pulse), a camera (chosen to provide higher resolution and frame rate than the average smartphone used in our context), and an analysis software that is available open-source within MATLAB. The specific components include a GoPro Hero 8 camera, a 300-milliwatt (mW) laser with a wavelength of 532 nm, optical components, Conduct-O-Fil (Silver-coated Hollow Ceramic Spheres) as tracer particles, and the open-source PIVLab app in MATLAB as the analysis software. The relatively low power of the laser enabled significant cost reduction but also limited the physical size of the flow phenomena that could be investigated. Therefore, this system is best for integration into any flow generation setup of a benchtop size. The system requires a supply voltage of only 15 V, which can be achieved with a standard desktop power supply unit. Laser pulsing is also possible with this system, leveraging a microcontroller. The camera can be operated at different frame rates, enabling the system to capture flows across a range of Reynolds numbers (***Re***). This dimensionless parameter expresses the ratio of inertial to viscous effects in the flow, hence capturing whether a flow is laminar (relatively low values), turbulent (relatively high values), or transitioning between the two [2]. The equation gives it:

$$Re=\frac{\rho VD}{\mu }$$

Where ρ is the density of the fluid, ***V*** is the velocity of the flow, ***D*** is the diameter of the flow, and μ is the dynamic viscosity of the fluid. This system can be used for visualizing fluid flows that operate in relatively low to medium Reynolds numbers, that is, laminar to transitional flows. In order to capture flows at higher Reynolds numbers, the GoPro camera can be replaced with a camera with a higher frame rate, less noise, and resolution, enabling the capture of the respective particle motions more accurately [25], [16].

## 2 Hardware description

**Fig. 1:**
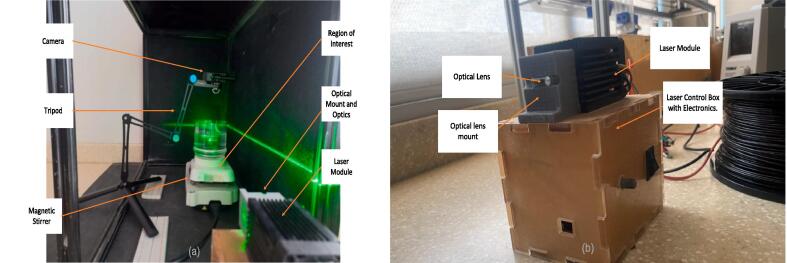
Two views of the total PIV System, with key components labeled.

This PIV system is as low cost as it is modular, meaning that the components can easily be upgraded as the user desires and the available budget increases. Fig. 1 shows two views of the PIV system, and Table 1 captures the system's specifications. The components chosen are available from many different suppliers, making the creation of this system feasible. The camera has a 1920 x 1080 pixels resolution at 120 frames per second [26]. Conduct-O-Fil serves as the tracer particle seeded in the flow, which is illuminated to visualize the motion of the fluid. These are neutrally buoyant; hence, the particles have a density very close to that of water. The laser module and optical lens create a 2-D light sheet with an area exceeding 0.25 cubic meters (Table 2).

**Table 1:** PIV system component specifications.

**Component**	**Specifications**
Camera (GoPro Hero 8)	https://doi.org/10.17632/cgmmttr4bz.2
532 nm laser module (green laser)	300 mW with TLL
Cylindrical optical lens	5x12 mm
Tracer particles	Conduct-O-Fil® AG-SL150-30-TRD Silver-coated Hollow Ceramic Spheres

**Table 2:** Design files summary.

**Design file name**	**File type**	**Open-source license**	**Location of the file**
1125T521_Cdx Grade Plywood Sheet.SLDPRT	CAD	CC BY4.0	https://doi.org/10.17632/cgmmttr4bz.2
2000 MW 450 nm Blue Laser.STEP	CAD	CC BY4.0	https://doi.org/10.17632/cgmmttr4bz.2
2020_int_L_joint.STEP	CAD	CC BY4.0	https://doi.org/10.17632/cgmmttr4bz.2
Al2020_T_Slot.SLDPRT	CAD	CC BY4.0	https://doi.org/10.17632/cgmmttr4bz.2
Box parts v5.step	CAD	CC BY4.0	https://doi.org/10.17632/cgmmttr4bz.2
go10 d2.SLDPRT	CAD	CC BY4.0	https://doi.org/10.17632/cgmmttr4bz.2
Lens_Mount.SLDPRT	CAD	CC BY4.0	https://doi.org/10.17632/cgmmttr4bz.2
Lens.SLDPRT	CAD	CC BY4.0	https://doi.org/10.17632/cgmmttr4bz.2
LensAssem.SLDASM	CAD	CC BY4.0	https://doi.org/10.17632/cgmmttr4bz.2
TestSection.SLDPRT	CAD	CC BY4.0	https://doi.org/10.17632/cgmmttr4bz.2
TRIPOD BASE QUICK.stp	CAD	CC BY4.0	https://doi.org/10.17632/cgmmttr4bz.2
Tripod Base.SLDPRT	CAD	CC BY4.0	https://doi.org/10.17632/cgmmttr4bz.2
Tripod Leg.SLDPRT	CAD	CC BY4.0	https://doi.org/10.17632/cgmmttr4bz.2
tripod_base_for use.SLDPRT	CAD	CC BY4.0	https://doi.org/10.17632/cgmmttr4bz.2
tripod_n_gopro.SLDASM	CAD	CC BY4.0	https://doi.org/10.17632/cgmmttr4bz.2
PIV System.SLDASM	CAD	CC BY4.0	https://doi.org/10.17632/cgmmttr4bz.2
PIVbox_accepted.dxf	DXF	CC BY4.0	https://doi.org/10.17632/cgmmttr4bz.2
Laser_pulse.ino	Code	CC BY4.0	https://doi.org/10.17632/cgmmttr4bz.2
Pulsing_Circuit.kicad_sch	Schematic	CC BY4.0	https://doi.org/10.17632/cgmmttr4bz.2

The low-cost PIV system uses a camera to record flow data generated in the test section, illuminated by a pulsing laser light sheet. The flow data is then post-processed to obtain parameters such as vorticity and velocity magnitudes. The system allows for the following:

• Visualization tool for fluid flow experiments
• Quantitative measurement tool, leveraging cross-correlation of groups of particles across consecutive frames.
• Validation of Computational Fluid Dynamics (CFD) results
• Implementation of experiments in different fluids and orientations.
• Research and development of new products by testing their behavior in a controlled environment.

## 3 Design files summary

The files used to make fabricate the PIV system are shown in Table 2, Table 3 below. The files can be found in a Mendeley data repository which can be accessed by the links found in Table 2. Table 3 provides are description of the files for easy replication of the PIV system.

**Table 3:** Design file description.

**Design file name**	**Descriptions**
1125T521_Cdx Grade Plywood Sheet.SLDPRT	This is the plywood used to enclose the regions of the test section to ensure proper observation of the region of interest.
2000 MW 450 nm Blue Laser.STEP	This device illuminates the test section to ensure optimal flow observation.
2020_int_L_joint.STEP	The L joint holds the aluminum extrusions together.
Al2020_T_Slot.SLDPRT	This is the aluminum extrude file for a 2020 aluminum extrusion.
Box parts v5.step	This is the file for the laser control box with all the holes in the box.
go10 d2.SLDPRT	This is the GoPro Hero CAD model that represents the camera for observation of the flow.
Lens_Mount.SLDPRT	This is the mount for the optical lens to create a 2D light sheet.
Lens.SLDPRT	This CAD model represents the optical lens used to create the 2D light sheet.
LensAssem.SLDASM	This is the SolidWorks assembly of the optical lens mounted into the mount.
TestSection.SLDPRT	This is the frame of the test section using the aluminum extrusion parts.
Tripod Base.SLDPRT	This is the base of the GoPro tripod, which was used in the camera assembly.
Tripod Leg.SLDPRT	This is the leg of the GoPro tripod. It is used in the tripod and GoPro assembly.
tripod_n_gopro.SLDASM	This is the assembly of the GoPro and the Tripod.
PIV System.SLDASM	This is the complete assembly of the Particle Image Velocimetry in SolidWorks.
PIVbox_accepted.dxf	This is the DXF file for laser cutting. This box contains the circuitry for powering and pulsing the laser.
Laser_pulse.ino	This is the code for pulsing the laser.
Pulsing_Circuit.kicad_sch	This is the schematic diagram that shows all the circuit connections. This can be opened with the KiCAD software, which is free to use and download.

## 4 Bill of materials summary

The total for the bill of materials sums up to $520.50. Note that this does not include the cost of 3D printing and laser cutting of the casing. Also note that the price of the tracer particles is an estimate and changes depending on the size of the order placed.

## 5 Build instructions

**Fig. 2:**
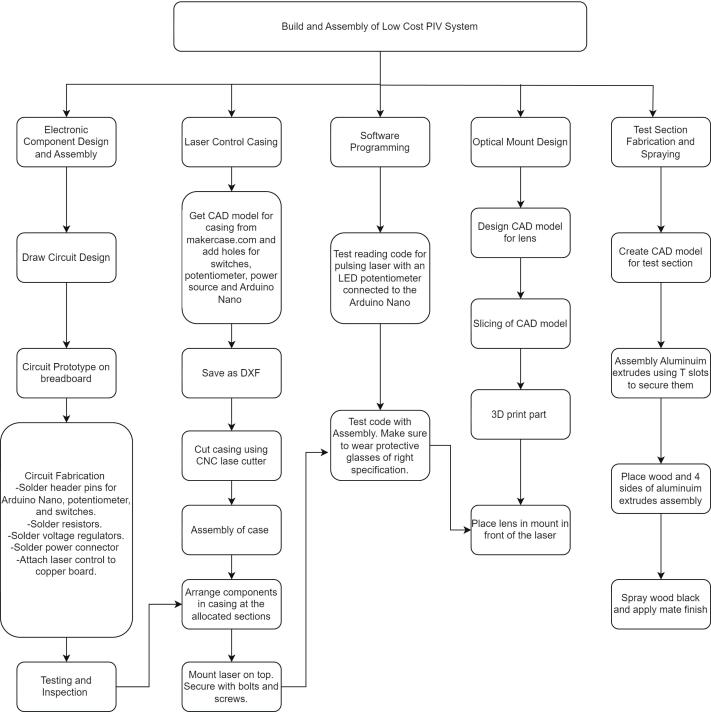
Flow diagram for build instructions.

The low-cost PIV system fabrication consists of seven streams of work: 1) electronic component design and assembly, 2) laser control casing, 3) software programming, 4) optics mount design, and 5) test section fabrication and spraying. 6) Tracer particles and 7) Post Processing software will be discussed in this section as well. Fig. 2 captures a flow diagram of the critical steps to realize these seven streams and complete the PIV system. The build starts with the electronic component design and assembly, which details how the control of the laser module is achieved. The next stream is the laser control casing, which involves designing and fabricating a case for electronics. Software programming is done after the build with the code intended to control the pulsing of the laser. Optics mount design details the process of making a mount for positioning the optical lens in front of the laser to generate a 2D light sheet. The test section encloses the region of interest to provide maximum contrast and visualization of tracer particles to allow effective postprocessing (Table 3).

The 300mW laser used in this paper is a class 3 laser, which has the potential to cause medium-level eye injury such as vision loss, painless eye injury, loss of acuity, blind spot, and retinal burn but poses minimal danger to the skin [27], [28]. Hence operation of this system should never be carried out without all personnel present in the room donning appropriate personal protective eyewear (PPE). Standard protective eyewear is not sufficient and rather laser safety goggles must be used. All laser safety goggles are labeled with the wavelength range that they cover and their level of optical density (OD) [28], [29]. The user must ensure that the goggles selected match the wavelength of the laser being used and note that the higher the OD value, the more protection it offers. A suitable option for this laser (532 nm) is provided in Table 4.

**Table 4:** BOM summary.

**Designator**	**Component**	**Number**	**Cost per unit –Dollars ($)**	**Total cost −** **Dollars ($)**	**Source of materials**	**Material type**
Laser module	12 V DC 532 nm 300mW green DPSS laser module	1	$79.99	$79.99	300mW 532 nm Green DPSS Laser Module with TTL Continuous	Metal
Optical glass cylindrical lens	Lights88 Laser diode rod lens optical glass line laser cylinder lens 5 mm x 12 mm	1	$4.98	$4.98	lights88 Laser Diode Rod Lens Optical glass line laser cylinder lens 5x12mm 2pcs	Glass
GoPro Hero 8	GoPro Hero 8 Black	1	$256.49	$256.49	GoPro Hero8 Black	Semiconductor
Tripod stand	SOONSUN Extendable Selfie Stick Tripod Stand for GoPro Hero 12/11/10/9/8/7/6/5/4/3 GoPro Max DJI Osmo Action Insta 360 AKASO SJCAM, Portable Vlog Tripod Stand with Phone Mount for Smartphone	1	$12.99	$12.99	SOONSUN Extendable Selfie Stick Tripod Stand for GoPro Hero 12/11/10/9/8/7/6/5/4/3 GoPro Max DJI Osmo Action Insta 360 AKASO SJCAM, Portable Vlog Tripod Stand with Phone Mount for Smartphone	Metal
Arduino Nano v3	Nano V3.0 Micro Controller Module ATmega328P Module Nano Board CH340 USB Cable Compatible with Arduino Nano V3.0	1	$5.58	$5.58	For Nano V3.0 Micro Controller Module ATmega328P Module Nano Board CH340 USB Cable Compatible with Arduino Nano V3.0	Semiconductor
Resistors	100PCS 1/4W Carbon Film Resistors 5 % 1R-1 K ohm Color Ring Resistance	1 pack (2 resistors used)	$0.48	$0.48	100PCS 1/4W Carbon Film Resistors 5 % 1R-10 M 10R 47R 100R 220R 1 K 10 K 4 K7 100 K 560 K 1 M 3 M3 ohm Color Ring Resistance	Semiconductor
10 K variable resistor / Potentiometer	Potentiometer Resistance WH148 3Pin 10 K Ohms	1	$0.35	$0.35	5Pcs/lot Potentiometer Resistance WH148 3Pin 1 K 2 K 5 K 10 K 20 K 50 K 100 K 500 K 1 M Ohm Linear Taper Rotary Potentiometer Resistor	Semiconductor
5 Volts voltage regulator	10pcs LM7805 L7805 7805 Voltage Regulator IC 5 V 1.5A TO-220	1	$0.88	$0.88	10pcs LM7805 L7805 7805 Voltage Regulator IC 5 V 1.5A TO-220	Semiconductor
12 Volts voltage regulator	10PCS L7812CV TO-220 L7812 LM7812 7812 Positive-Voltage Regulators IC	1	$0.70	$0.70	10PCS L7812CV TO-220 L7812 LM7812 7812 Positive-Voltage Regulators IC	Semiconductor
Perforated copper board	7x9cm Double-Sided copper PCB board	1	$0.56	$0.56	2PCS 7x9cm Double Side Prototype PCB Board 7*9cm Universal Printed Circuit Board For Experimental PCB Copper Plate	Semiconductor
Female power jack	10Pcs 3A 12v For DC Power Supply Jack Socket Female Panel Mount Connector 5.5 mm 2.1 mm Plug Adapter 2 Terminal Types 5.5*2	1	$0.85	$0.85	10Pcs 3A 12v For DC Power Supply Jack Socket Female Panel Mount Connector 5.5 mm 2.1 mm Plug Adapter 2 Terminal Types 5.5*2	Semiconductor
Safety goggles	GOSONO Laser Safety Glasses 190 nm to 540 nm (Laser protective eyewear with velvet box, pack of 2)	2	$16.99	$33.98	Goggles Laser Safety Glasses 190 nm to 540 nm Laser protective eyewear With Velvet Box	Plastic
Tracer particles*	Potters Industries Conduct-O-Fil® AG-SL150-30-TRD Silver-coated Hollow Ceramic Spheres	1	$20	$20	Potters Industries Conduct-O-Fil® AG-SL150-30-TRD Silver-coated Hollow Ceramic Spheres	Polymer
Header pins	Header pins 1x 40	1	$0.24	$0.24	Female Pin header 1 × 40	Plastic and Metal
Switches	Electrical mini rocker switch with wire	2	$0.55	$1.10	1pcs Push button switch small car circuit wire speaker electrical mini rocker switch with wire	Plastic
Plexiglass	300x300mm plexiglass clear acrylic board, organic plastic sheet 3 mm glass methacrylate	1	$10.29	$10.29	200x200mm 300x300mm plexiglass clear acrylic board, organic plastic sheet, 2 mm, 3 mm and 4 mm glass methacrylate	Plastic
Aluminum extrudes	4 pcs 200 mm T Slot 2020 Aluminum Extrusion European Standard Anodized Linear Rail for 3D Printer Parts and CNC DIY Silver(7.874 in.)	1 set (4 pcs)	$11.99	$11.99	4pcs 150 mm T Slot 2020 Aluminum Extrusion European Standard Anodized Linear Rail for 3D Printer Parts and CNC DIY Silver(5.9 in.)	Metal
Plywood	Plywood Plank DIY Wood Material Supplies 0.2 cm	1 (5 pieces)	$4.47	$4.47	Multi Size Aviation Model Layer Board Basswood Plywood Plank DIY Wood Material Supplies	Organic
Black spray paint	Krylon K05613007 COLORmaxx Acrylic Latex Brush On Paint for Indoor/Outdoor Use, ½ Pint, Satin Black	1	$15.73	$15.73	Krylon K05613007 COLORmaxx Acrylic Latex Brush On Paint for Indoor/Outdoor Use, ½ Pint, Satin Black	Inorganic
Matte spray finish	Spray paint RAL-9005 matte black 200 ML	1	$10.00	$10.00	spray paint RAL-9005 matte black 200 ML	Inorganic
Slot nuts	20 Series Slot T-nut Sliding T Nut Hammer Drop In Nut Fasten Connector 2020 Aluminum Extrusion M5	1	$1.75	$1.75	10/20/50/100pcs M3/M4/M5*10*6 for 20 Series Slot T-nut Sliding T Nut Hammer Drop In Nut Fasten Connector 2020 Aluminum Extrusion	Metal
L Joints	10PCS T Slot L-Shape Aluminum Profile Interior Corner Connector Joint Bracket for 2020 EU Alu-profile with Screws	2	$3.63	$7.26	10PCS T Slot L-Shape Aluminum Profile Interior Corner Connector Joint Bracket for 1515 2020 3030 4040 EU Alu-profile with Screws	Metal
Hex bolts	M5 304 Stainless Steel Metric Thread DIN933 Outside Hex Head Bolt External Hexagon Head Screw	2	$2.42	$4.84	M3 M4 M5 M6 M8 A2-70 304 Stainless Steel Metric Thread DIN933 Outside Hex Head Bolt External Hexagon Head Screw	Metal
MATLAB	MATLAB Student License	1	$55.00	$55.00	MATLAB license	Software

Safety precautions should be taken when working with any electrical equipment, as there is always a risk of electrocution, which could cause muscle contractions and vertical fibrillation [30]. Although the risks associated with this system are minimal due to the low voltage being used, a few safety recommendations should be followed. First, ensure you do not touch exposed wires when operating any electrical equipment in this system. Second, electrical equipment should not be operated barefoot [31].

### 5.1 Electronic component design and assembly

**Fig. 3:**
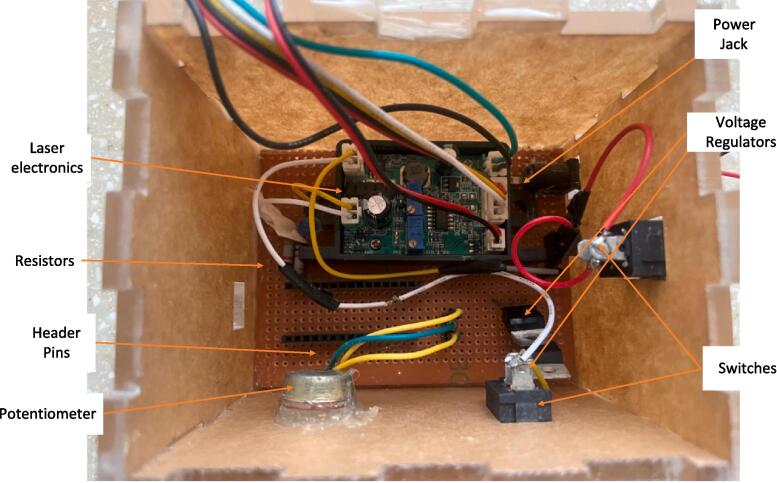
Electronics assembly, as viewed from the top of the opened casing.

**Fig. 4:**
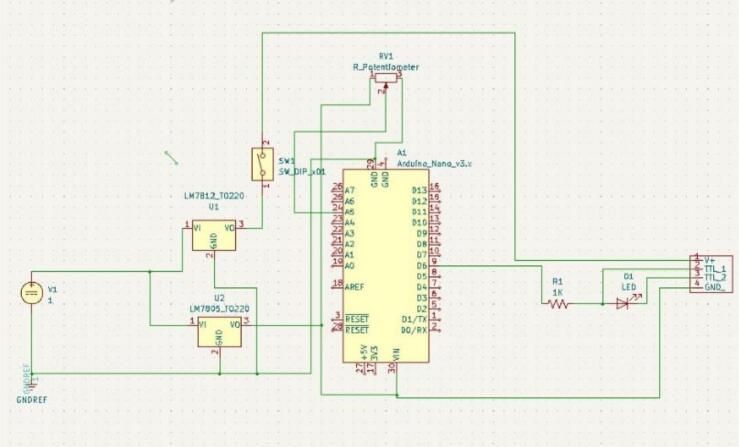
Electronics schematic.

Fig. 3 shows the electronics assembly used to control the power and pulsing of the laser module. The items needed include a power jack, two voltage regulators (LM7812 and LM7805), header pins, switches, resistors, a variable resistor (potentiometer), a perforated board, and the Arduino Nano (see Fig. 4 for the schematic diagram). The electronic components are soldered onto the perforated circuit board. The power jack connects to switches, which connect to voltage regulators. These regulators set the voltage to 5 V (using the LM7805) for the Arduino Nano, positioned on the header pins, and to 12 V (using the LM7812) for the laser module. All relevant files for this project can be found in the repository named pulsing_Circuit.kicad_sch. The schematic diagram was created using KiCAD 7.0, an open-source software for making electronic schematics, footprints, and PCBs.

### 5.2 Laser control casing

**Fig. 5:**
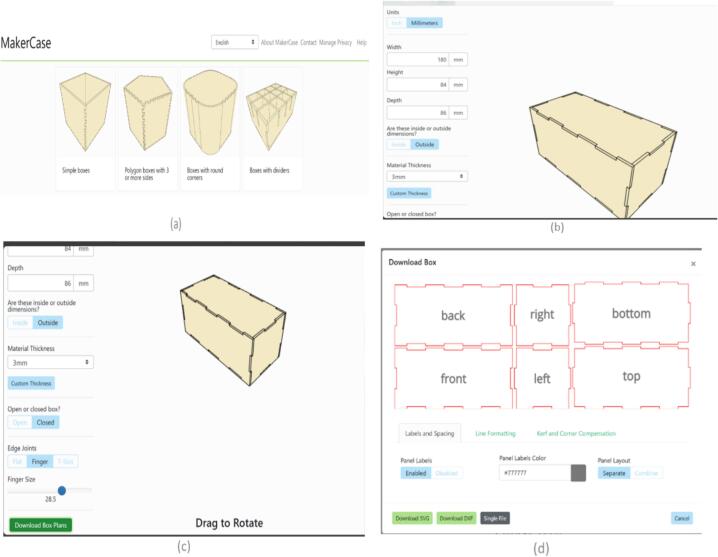
(a) Box type selection (b) Dimensions and Material Thickness (c) Box configuration using edge joints and finger size (d) Box parts for DXF download.

Makercase.com was chosen for ease of use in creating the laser control casing. The instructions are first to select the box type. Dimension the box appropriately using the width, height, and depth edit fields. Any update to these boxes can be seen visually within the 3D view as seen in Fig 5. The website allows the selection of the box's thickness and other configurations, such as the finger and the size of the fingers. Finally, click on the download box plans, download the DXF files, and edit them. The DXF file in the repository is saved as PIVbox_accepted.dxf. The box used was a simple closed box with dimensions of 180 mm by 84 mm by 86 mm with a material thickness of 3 mm using outside dimensions and finger edge joints with 28.5 finger size.

The DXF file is then imported into SolidWorks, and holes are added for mounting switches, a potentiometer, an Arduino cable, and a power cable. Import the DXF files into the BOSSLASER LaserCAD software and laser cut with the BOSSLASER cutter.

### 5.3 Software programming

The code for the system is written in C/C++ programming language using the Arduino Integrated Development Environment (IDE). The Arduino is free and easy to use, with example code to help beginners learn quickly. The code is for the system's response to the potentiometer change. The change in the potentiometer determines the laser pulse rate, ranging from 0 Hz pulse to 120 Hz pulse. There are four levels of laser pulse rate: 0 Hz, 30 Hz, 60 Hz and 120 Hz. The potentiometer is connected to an analog pin of the Arduino nano (labeled A0 to A5 on the board). Pin A0 was used, and the analog input into the Arduino nano was mapped to a value between 0 and 1023, dividing 1023 into four even ranges of our four pulse rates stated above. The range of values the potentiometer is in will map to a specific pulse rate. The code for the system is in Laser_pulse.ino file.

### 5.4 Optics mount design

**Fig. 6:**
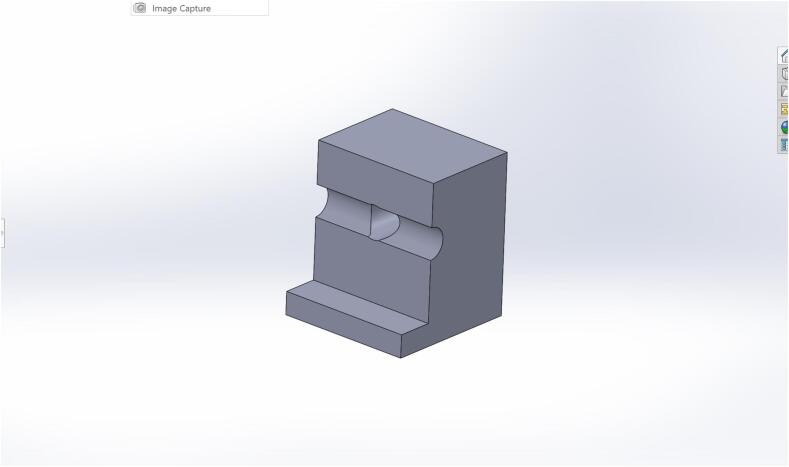
Optical Mount.

The CAD model of the optical lens mount is modeled in SolidWorks. The optical mount is designed with simplicity and repeatability in mind. The lens slots perfectly into position and is directly in line with the light sheet created by the laser. Precautions must be taken when mounting the optical lens before the laser. Ensure PPE is worn before positioning the mounted optical lens in front of it. Adjust the mount position until a faint line appears in the test section. Fig. 6 shows a picture of the CAD rendition of the mount. The file can be found in the repository under the name Lens_Mount.SLDPRT. This file must be converted to a.stl or 0.3mf file from SolidWorks and imported into a slicer software such as Ultimaker Cura or Prusa Slicer before it can be 3D printed. These softwares are open source and free to use with the exception of SolidWorks for which Autodesk Fusion 360 or FreeCAD can be used as alternatives. The individual part files are also included in the repository. The filament used for printing was PLA, and the printer was the Prusa i3. An Ender 3 Pro or Ender v2 can also be used to print the mount.

### 5.5 Test section

**Fig. 7:**
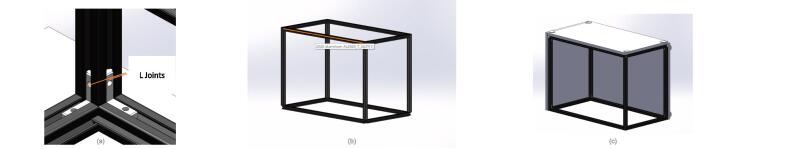
A) aluminum extrudes joining using l joints b)assembled frame of aluminum extrudes c) test section assembly with wood over aluminum extrudes note: the test section walls are shown in grey here to distinguish them from the rest of the assembly easily; however, the walls should be painted black to ensure maximum light absorption.

The test section created here is an open box that serves to house the test object and reduce the effect of ambient airflow over it. It was designed as a simple assembly of aluminum extrusions and wood sheets. The wood was coated with black paint and given to a matte finish using a matte spray. The black color was used to reduce the amount of external light entering the test section, enabling more excellent contrast between the illuminated tracer particles and the background. The dimensions of the test section are 61.5 x 42 cm x 35 cm. The test section components are the 20 mm-by-20 mm aluminum extrusion, the L joints, slot nuts, hex bolts, plywood, black paint, and the matte finish spray. The 20 mm-by-20 mm aluminum extrusions are held together using the L joints, as shown in Fig. 7, which have headless screws that can be tightened using an appropriately sized Allen key. The black paint was applied to the aluminum extrusion and the plywood. The plywood was fastened to the aluminum extrusions using hex bolts screwed into the slot nuts. The CAD file was saved in the repository with the name TestSection.SLDPRT. The complete test section is in the overall assembly, named PIV System.SLDASM.

### 5.6 Tracer particles

Tracer particles (seeding particles) are an essential part of a PIV system. Carrying out experiments to figure out the fluid flow properties, such as velocity vector fields, requires visible particles that can be traced as the flow moves [32]. Typically, tracer particles are neutrally buoyant and very small, usually micrometers in diameter. Potters Industries' Conduct-O-Fil AG-SL150-30-TRD silver-coated hollow ceramic spheres were the tracer particles used to validate this system. This is a fiberglass filler material, hence was cheaper than custom-made PIV tracer particles. The particle size is 100 µm, and the density is 1.0 g/cc [33]. This material is neutrally buoyant in water, and its silver coating scatters the laser light well, hence it was deemed a good fit here.

### 5.7 Post-processing software

Typically, post-processing of the video or images is required for a PIV system. Post-processing software is used to accomplish the task of obtaining information such as the velocity field vectors, vorticity and so on. This software usually uses fast Fourier transform (FFT) algorithms to compute the cross-correlation required to obtain velocity data [34]. In this paper, a software known as PIVLab was used. PIVLab is a software package in MATLAB and thus requires MATLAB to run. Other postprocessing software exists, such as OpenPIV, which is mainly Python-based, and mI-PIV, which is smartphone-based. PIVLab was chosen for this paper because it has a better and more responsive user interface than OpenPIV and mI-PIV from testing. OpenPIV has a more prominent supporting community and better documentation, making it a good option as well. mI-PIV is user-friendly but mainly provides visual data.

Download the PIVLab App for MATLAB from the MathWorks website: https://www.mathworks.com/matlabcentral/fileexchange/27659-pivlab-particle-image-velocimetry-piv-tool-with-gui. For more information on using the App; there is a playlist of instructional videos by the creator that can be found here: https://youtu.be/g2hcTRAzBvY [35].

**Fig. 8:**
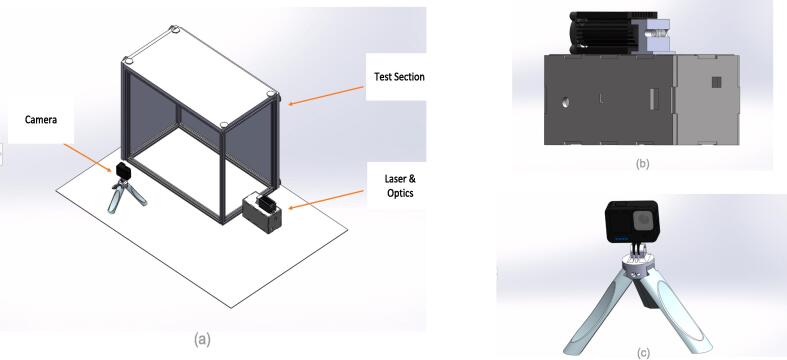
PIV setup (a) Full Setup (b) Laser & Optics (c) Camera.

The final build should resemble what is depicted in Fig. 8.

To set up an experiment, position the camera perpendicular to the laser module and optics, as depicted in Figs. 1 and 8. Ensure the laser module and optics setup are directed straight into the test section. The laser module should be placed onto its control casing containing components such as the Arduino Nano and voltage regulators. As stated above, the optics are mounted in front of the laser module.

Before conducting the experiment, wearing personal protective equipment (PPE) is essential. Once ready, place the experimental setup in the test section and activate the laser and optics. The camera should then be positioned correctly to capture the experiment effectively, ensuring that the flow dynamics are adequately recorded for postprocessing in the PIVLab software.

## 6 Operation instructions

**Fig. 9:**
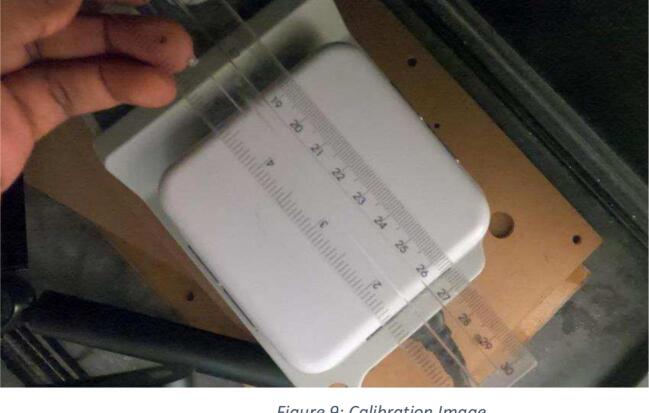
Calibration Image.

NOTE: Depending on the time of flow being observed, the user might have to turn on additional equipment. For example, analyzing the rotating water flow in a magnetic stirrer will require turning on the magnetic stirrer. Always remember to wear protective glasses.

**Fig. 10:**
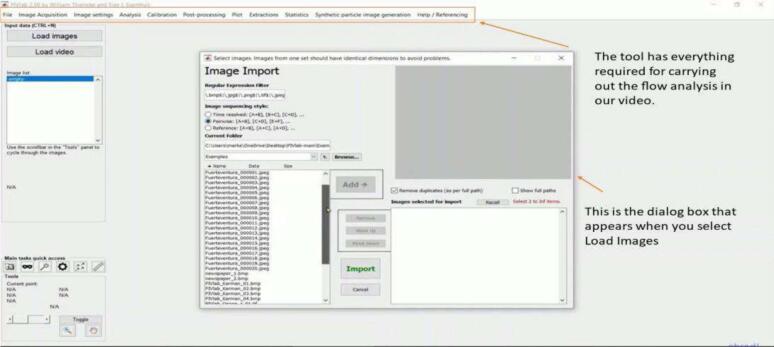
Image of Video Import.
  b. Select the region of interest in the Image by selecting exclusions from the image settings tabs. Note: This may not be needed depending on the type of analysis.
  c. Next, select Image pre-processing from the Image settings tab. This step helps emphasize the particles to analyze (Fig. 11).

a) Orient the laser module such that the beam points into the test section.
b) Mount the camera perpendicular to the laser module and face into the test section from the other open end.
c) Place the object of study into the test section.
d) Place the lens mount to create a light sheet in the desired orientation (either vertically or horizontally, depending on the kind of study being performed).
e) Wear protective glasses to protect the eyes.
f) Turn on the laser by flipping the switch on the laser control box connected to a 15-volt power source.
g) Induce the flow to be observed by ensuring the light sheet illuminates tracer particles in the region of interest.
h) Turn on the camera to record the results.
i) Take a calibration image using a ruler, as shown in Fig. 9.

j) Import the video in PIVLab and carry out the analysis as follows.
  a. Import the video or Image into PIVLab by clicking the Load Video or Load Image Button (Fig. 10).

**Fig. 11:**
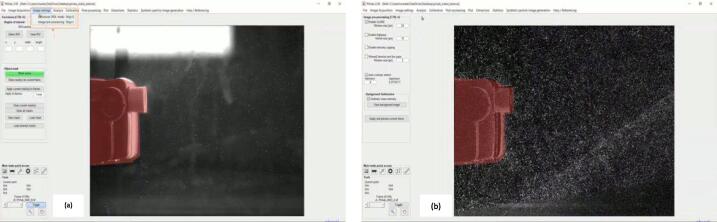
Image settings and using exclusions, Image pre-processing to highlight particles [36].
  d. Select PIV settings from the Analysis tab. Here, set the resolution for the analysis to enable the software to identify the particles in the video. This will be helpful as the video changes frames.
  e. Select PIV settings from the Analysis tab. Here, the resolution is set for the analysis to enable the software to identify the particles in the video, which will be helpful as the video changes frames.
  f. Next, select ANALYZE! from the Analysis tab and run the analysis to create the vectors. NOTE: this may take a while (Fig. 12).

**Fig. 12:**
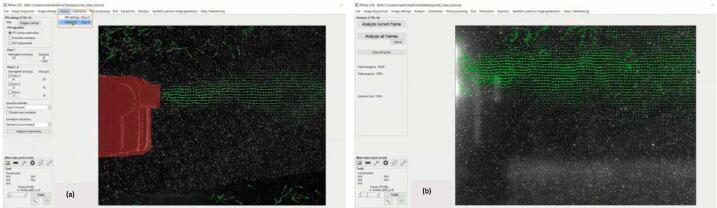
PIV settings, analyzing all the frames [36].
  g. After analysis, upload a calibration image to set the reference frame and give physical units to the pixels. Set the distance in mm and the time step on the Image's left side. Under Setup Offsets, set the reference frame with the direction to the right and up as positive for x and y, respectively (Fig. 13).

**Fig. 13:**
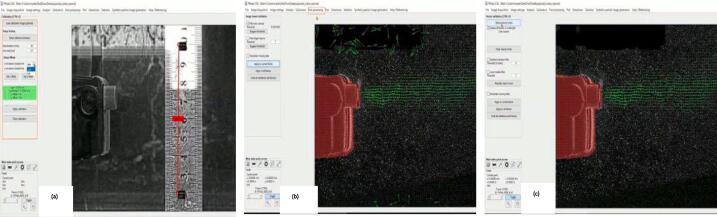
Image calibration, setting physical units and reference frame, Image Validation, Setting Velocity limits [36].

**Fig. 14:**
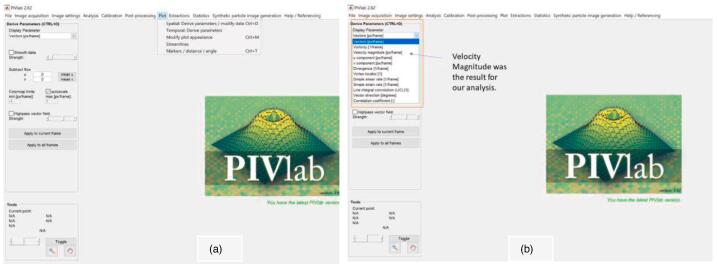
(a) The plot tab items. (b) Selecting velocity magnitude.

Next, postprocessing is used to clean up the results and give meaning to vectors by applying calibration to the frames.

h. Finally, click on the Plot tab and select the results to plot. In this case, velocity magnitude is the desired result. Go to Derivative Parameters from the Plot Tab, select velocity magnitude from the Display Parameter drop, and click Apply to frames (Fig 14).

## 7 Validation and characterization

**Fig. 15:**
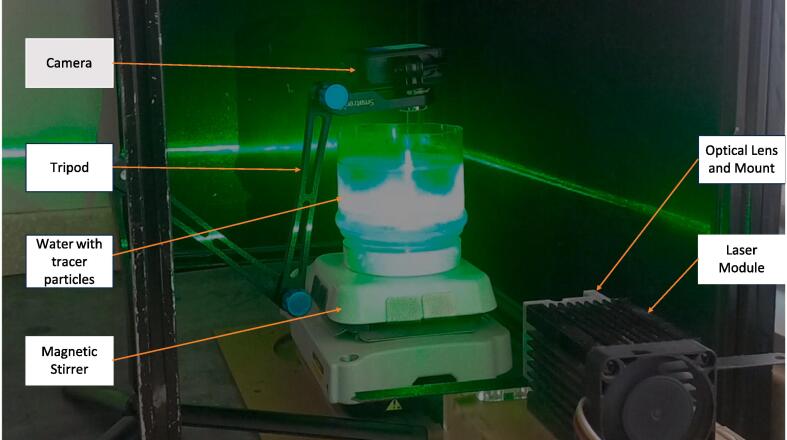
Rotating flow experiment.
The calibration image was recorded with the camera.
The results were processed using PIVLab in MATLAB.
Data analysis in the software was conducted, following the instructions in section 6 above.

A validation experiment was conducted to test this hardware. A magnetic stirrer in a beaker of water created a rotating flow. The aim was to compare the measurements obtained from this low-cost PIV system to the results expected. Specifically, the maximum velocity measured was compared to the velocity set as the input on the magnetic stirrer. The following was done to create the experiment:

Some Conduct-O-Fil was stirred in a measuring cylinder filled with water.
The stirrer bar was placed in the measuring cylinder.
The setup was placed on a magnetic stirrer, and revolutions per minute (rpm) were set on the stirrer.
A light sheet was generated on the water's surface to illuminate the tracer particles.
The camera (GoPro Hero 8) was mounted perpendicular to the light sheet. A frame rate of 120 and a resolution of 1080p was used.
Safety glasses were worn to protect the eyes from laser light.
The laser was turned on, and a video of the rotating flow was observed and recorded, as seen in Fig. 15.

The setup in Fig. 15 shows the laser module with the optics positioned to create a horizontal light sheet on the water's surface. The camera was placed above the flow to view the water's surface illuminated by the light sheet.

### 7.1 Characterization results

The magnetic stirrer was run at three rpm values: 60, 92.5, and 125. The experiment at each rpm value was carried out three times to determine the repeatability of readings. The analysis was carried out in PIVLab following the steps presented in section 6. The lower RPM values were observed to be ideal for this setup, as they avoided funneling development within the system. Funneling is the phenomenon in which a funnel-like shape develops from the center of rotation of the magnetic stirrer toward the surface. This creates a non-uniformity in the velocity profile along the system's height, making calculating velocity values on the surface challenging to estimate [37]. The funneling phenomena showed signs of beginning to form at the highest rpm value tested (125).

The parameter of interest for this experiment was the velocity magnitude at the surface, along the radius of the stirrer. The velocity magnitudes of the fluid particles (**V**) were computed analytically using the formula **V** =πDN, where **N** is the revolutions per second, and **D** is the length of the magnetic stirrer bar. This was compared to the velocity magnitude calculated in the PIVLab results, selecting points located directly on the circle's radius corresponding to the length of the magnetic stirrer bar (2.8 cm). Note that the Image used for these calculations in the software was the mean Image, which showed the average velocity value at each respective point over all of the frames captured by the camera.

**Fig. 16:**
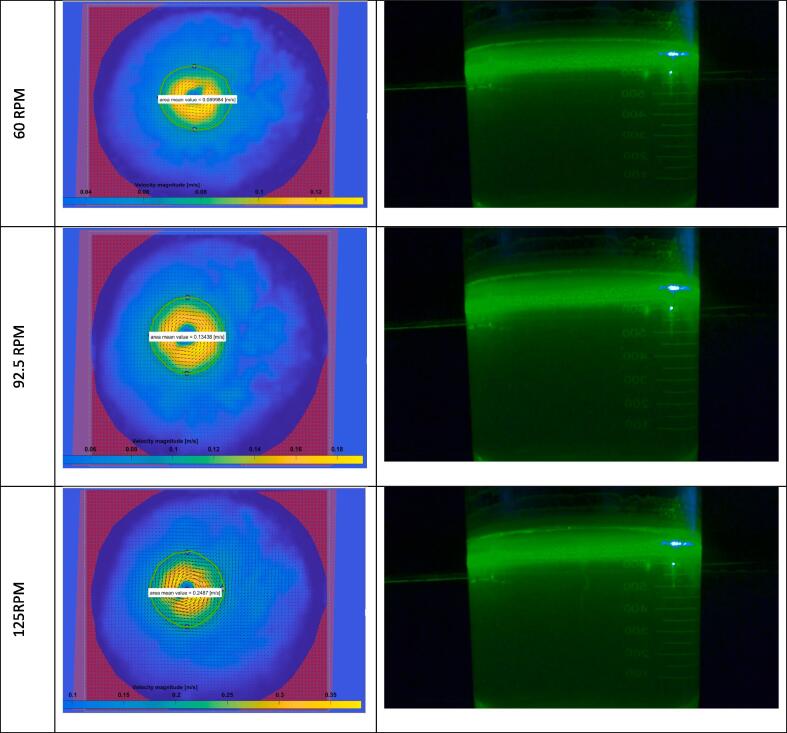
(Left) Velocity field generated in PIVLab and (Right) Side View of the actual system at each of the three rpm values tested. At the highest rpm value, signs of funneling begin to appear.

Fig. 16 shows a side-by-side representation of the velocity magnitude values generated by PIVLab and a side view of the flow field in motion. Each row depicts results from one rpm value. The mean velocity magnitude of the flow was taken at the circle (shown in green) where the length of the stirrer bar reached. The three rpm values tested —60 rpm, 92.5 rpm, and 125 rpm translate to 1.00, 1.54, and 2.05 in seconds, respectively. The mean velocity magnitudes measured by the PIV system at the different RPMs were 0.0900, 0.1344, and 0.2487 m/s, respectively. When computed analytically, these values were expected to be 0.0880, 0.1356, and 0.1833 m/s. Hence, the percentage difference between these results was 2.30 %, 0.91 %, and 35.71 % respectively. Funneling began to develop at the highest RPM value, as seen through a small streak emerging from the surface downwards. Hence, the expected value began to deviate from that which can be computed from the basic model of a perfectly 2-D rotating flow, and therefore, the percentage difference increased. For the lower two values, this percent difference was minimal, revealing the reliability of this PIV system in determining expected values for flows that the system can readily capture in 2-D. Furthermore, the system was found to be consistent, as the three iterations of the experiment at each rpm differed no more than 6 % from each other.

## 8 Conclusion

The teaching and research of fluid mechanics benefit substantially from leveraging hardware to visualize and measure flow phenomena. Hence, there is a need for low-cost alternatives for existing fluid mechanics experimental equipment. A low-cost Particle Image Velocimetry system was built at an institution in Ghana using off-the-shelf materials and widely available digital fabrication tools. The system comprises a camera, a laser module and optics, tracer particles, and a test section. The total cost of the system was about $500, and the authors look forward to further reducing its cost in future work, leveraging even more cost-effective components such as the Raspberry Pi camera.

The system was validated using a series of tests with a magnetic stirrer generating a rotational flow in a beaker. The system measured the resulting velocity magnitudes and was comparable to the results expected analytically, so long as the flow remained 2-D (no funneling). This system will be integrated into other facilities at the authors' institution to leverage it to study a broader range of flow fields, such as measuring lift and drag over airfoils and studying the wakes formed around bluff bodies. The availability of this platform opens the opportunity for universities and institutions across Sub-Saharan Africa and the Global South, more broadly, to lean into experimentation in fluid mechanics.

## 9 Ethics statements

The authors declare that they complied with the ethical guidelines of HardwareX and that they do not have any external influences that could have affected this work.

## CRediT authorship contribution statement

**Frederick Kojo Chaway Acquah:** Writing – review & editing, Writing – original draft, Visualization, Validation, Supervision, Software, Methodology, Investigation, Formal analysis, Data curation, Conceptualization. **Jeremiah Paul Konadu Takyi:** Validation, Software, Project administration, Methodology, Investigation, Conceptualization. **Heather R. Beem:** Writing – review & editing, Validation, Supervision, Project administration, Funding acquisition, Conceptualization.

## Declaration of competing interest

The authors declare the following financial interests/personal relationships which may be considered as potential competing interests: Frederick Kojo Chaway Acquah reports financial support was provided by Ashesi University. Frederick Kojo Chaway Acquah reports a relationship with Ashesi University that includes: employment and funding grants. Frederick Kojo Chaway Acquah has patent pending to CC By 4.0. If there are other authors, they declare that they have no known competing financial interests or personal relationships that could have appeared to influence the work reported in this paper.
